# Four years on: Pregnancy and birth outcomes reported in the MSBase pregnancy, neonatal outcomes, and Women’s Health Registry (2020–2024)

**DOI:** 10.1177/13524585251349125

**Published:** 2025-07-07

**Authors:** Vilija G Jokubaitis, Raed Alroughani, Ayse Altintas, Sara Eichau, Stella Hughes, Barbara Willekens, Dana Horakova, Eva Kubala Havrdova, Serkan Ozakbas, Cavit Boz, Mario Habek, Tomas Kalincik, Izanne Roos, Masoud Etemadifar, Marek Peterka, Jeannette Lechner-Scott, Jose E Meca-Lallana, Zuzana Rous, Jana Houskova, Alexandre Prat, Marc Girard, Radek Ampapa, Katherine Buzzard, Olga Skibina, Nevin A John, Allan G Kermode, Marzena J Fabis-Pedrini, Matteo Foschi, Andrea Surcinelli, Yolanda Blanco, Seyed Mohammad Baghbanian, Oliver Gerlach, Richard Macdonell, Zbysek Pavelek, Pavel Stourac, Pamela McCombe, Guy Laureys, Helmut Butzkueven, Anneke van der Walt, Orla Gray

**Affiliations:** Department of Neuroscience, School of Translational Medicine, Monash University, Melbourne, VIC, Australia; Department of Neurology, Alfred Health, Melbourne, VIC, Australia; Al Amiri Hospital, Sharq, Kuwait; Koc University School of Medicine and Koc University Research Center for Translational Medicine, Istanbul, Turkey; Hospital Universitario Virgen Macarena, Sevilla, Andalucía, Spain; Royal Victoria Hospital, Belfast, UK; Department of Neurology, Antwerp University Hospital, Edegem, Belgium; Department of Neurology and Center of Clinical Neuroscience, First Faculty of Medicine, Charles University in Prague and General University Hospital, Prague, Czech Republic; Department of Neurology and Center of Clinical Neuroscience, First Faculty of Medicine, Charles University in Prague and General University Hospital, Prague, Czech Republic; Izmir University of Economics, Medical Point Hospital, Izmir, Turkey; Multiple Sclerosis Research Association, Izmir, Turkey; Department of Neurology, Medical Faculty, Karadeniz Technical University, Trabzon, Turkey; Department of Neurology, University Hospital Center Zagreb, Zagreb, Croatia School of Medicine, University of Zagreb, Zagreb, Croatia; Neuroimmunology Centre, Department of Neurology, Royal Melbourne Hospital, Melbourne, VIC, Australia; CORe, Department of Medicine, University of Melbourne, Melbourne, VIC, Australia; Neuroimmunology Centre, Department of Neurology, Royal Melbourne Hospital, Melbourne, VIC, Australia; CORe, Department of Medicine, University of Melbourne, Melbourne, VIC, Australia; Faculty of Medicine, Isfahan university of Medical sciences, Isfahan, Iran; Faculty of Medicine and University Hospital in Pilsen, Charles University in Pilsen, Plzen, Czech Republic; Hunter Medical Research Institute, University of Newcastle; Department of Neurology, Hunter New England Health, John Hunter Hospital, Newcastle, NSW, Australia; Multiple Sclerosis CSUR, Clinical Neuroimmunology Unit, Neurology Department, Virgen de la Arrixaca Clinical University Hospital, IMIB-Arrixaca and NICEM Cathedra-UCAM-Catholic University San Antonio, Murcia, Spain; Faculty of Medicine, Palacky University and University Hospital Olomouc, Czech Republic; Hospital Ceske Budejovice, Czech Republic; CHUM MS Center and Universite de Montreal, Montreal, QC, Canada; CHUM MS Center and Universite de Montreal, Montreal, QC, Canada; Nemocnice Jihlava, Jihlava, Czech Republic; Department of Neurosciences, Eastern Health Clinical School, Box Hill Hospital, Monash University, Box Hill, VIC, Australia; Department of Neurology, Alfred Health, Melbourne, VIC, Australia; Department of Neurosciences, Eastern Health Clinical School, Box Hill Hospital, Monash University, Box Hill, VIC, Australia; Department of Medicine, School of Clinical Sciences, Monash University, Clayton, VIC, Australia; Department of Neurology, Monash Health, Clayton, VIC, Australia; Perron Institute for Neurological and Translational Science, University of Western Australia, Nedlands WA, Australia; QEII Medical Centre, Perth, Australia; Personalised Medicine Centre, Health Futures Institute, Murdoch University, Murdoch, WA, Australia; Institute for Immunology and Infectious Diseases, Murdoch University, Perth, WA, Australia; Perron Institute for Neurological and Translational Science, University of Western Australia, Nedlands WA, Australia; QEII Medical Centre, Perth, Australia; Personalised Medicine Centre, Health Futures Institute, Murdoch University, Murdoch, WA, Australia; Department of Neuroscience, MS Center, Neurology Unit, S. Maria delle Croci Hospital of Ravenna, Ravenna, Italy; Department of Biotechnological and Applied Clinical Sciences, University of L’Aquila, L’Aquila, Italy; Department of Neuroscience, MS Center, Neurology Unit, S. Maria delle Croci Hospital of Ravenna, Ravenna, Italy; Center of Neuroimmunology, Service of Neurology, Hospital Clinic de Barcelona, Barcelona, Spain; Neurology Department, Booalisina Hospital, Faculty of Medicine, Mazandaran University of Medical Sciences, Sari, Iran; Academic MS Center Zuyd, Department of Neurology, Zuyderland Medical Center, Sittard-Geleen, the Netherlands; Department of Neurology, School for Mental Health and Neuroscience, Maastricht University Medical Center, Maastricht, the Netherlands; Department of Neurology, Austin Health, Melbourne, VIC, Australia; Faculty of Medicine and University Hospital Hradec Kralove, Charles University in Prague, Hradec Kralove, Czech Republic; Masaryk University Brno and University Hospital, Brno, Czech Republic; Department of Neurology, the University of Queensland, Brisbane, QLD, Australia; Department of Neurology, University Hospital Ghent, Brussels, Belgium; Department of Neuroscience, School of Translational Medicine, Monash University, Melbourne, VIC, Australia; Department of Neurology, Alfred Health, Melbourne, VIC, Australia; Department of Neuroscience, School of Translational Medicine, Monash University, Melbourne, VIC, Australia; Department of Neurology, Alfred Health, Melbourne, VIC, Australia; South Eastern HSC Trust, Belfast, UK

**Keywords:** Multiple sclerosis, neuromyelitis optica spectrum disorder, pregnancy, disease-modifying therapy, neonatal outcomes

## Abstract

**Background:**

Family planning is an important aspect of multiple sclerosis (MS), and neuromyelitis optica spectrum disorder (NMOSD) management. Knowledge gaps remain, including optimal perinatal management strategies, and fetal risks associated with disease-modifying therapy (DMT) exposure.

**Objective:**

To describe perinatal DMT use, together with pregnancy and neonatal outcomes prospectively recorded in the International MSBase Pregnancy and Women’s Health Registry.

**Methods:**

We report summary statistics for data collected between May 2020 and August 2024.

**Results:**

A total of 1887 relapsing-remitting MS (RRMS), 12 primary-progressive MS (PPMS), 2 radiologically isolated syndrome (RIS) and 21 NMOSD completed pregnancies were recorded, including 1644 (85.5%) live births, 208 (10.8%) miscarriages, and 6 (0.3%) neonatal deaths. Most women had unassisted (53.8%) or assisted (7.4%) vaginal births. Seventy five percent of pregnancies had DMT exposures within 6 months preconception; 19% of NMOSD, and 62% of MS pregnancies were DMT-exposed during gestation; 18.1% of pregnancies reported in-pregnancy monoclonal antibody DMT exposure. No overt safety signals were seen.

**Conclusion:**

This first report from the newly launched MSBase pregnancy registry, establishes an increasing number of pregnancies being conceived on monoclonal antibody therapies. Although no safety signals were observed, it is important to continue monitoring for safety signals in real-world databases as the use of highly effective therapies continues to increase perinatally.

## Introduction

Women are disproportionately diagnosed with multiple sclerosis (MS) and related disorders. Women with MS outnumber men by three-fold,^1^ whereas women with neuromyelitis optica spectrum disorder (NMOSD) outnumber men by nine-fold.^2^ Commonly diagnosed in their third to fifth decades of life, most women will use disease-modifying therapies (DMTs) for much of their lives. Our understanding of the impact of these conditions, and the use of DMT on women’s health, including fecundity, fertility,^3^ and pregnancy,^2,4^ together with reproductive aging and menopause^3,5^ is evolving.

The evidence base for the safe and effective use of DMTs perinatally in women with MS is growing, increasing the confidence of neurologists to recommend and prescribe these medications during pregnancy planning and, for some DMTs, in pregnancy. Data from large registries including MSBase,^6,7^ the Canadian,^8^ Danish,^9^ French,^10^ German,^11,12^ Italian,^13^ and American^14^ pregnancy registries, as well as purpose-designed studies^15^ provide critical real-world insights that, in addition to post-marketing pharma-led studies, inform clinical guidelines on the use of these DMT through pregnancy and postpartum.^4,16^ The evidence base around NMOSD and NMOSD DMT use during pregnancy still remains limited, as 2000 exposed pregnancies are ideally required for each DMT to accurately define risks, including rare birth defects.^2,17–19^

While the international MS outcomes registry MSBase^20^ has prospectively collected pregnancy-related information since 2004, detailed pregnancy and neonatal outcomes data were not recorded. With the growing number of DMT available to treat MS and NMOSD, the need to capture more detailed data is evident. In response, MSBase launched a prospective Pregnancy, Neonatal Outcomes and Women’s Health Registry^21^ in 2020. The purpose of this registry is to address the impact of MS and related disorders, together with the impact DMTs on women’s health throughout the lifespan.^21^ In this first update from our women’s health registry since its launch, we specifically report on pregnancy and birth outcomes in the context of an expanding number of DMT available for the management of MS and NMOSD through pregnancy.

## Methods

### The MSBase Pregnancy, Neonatal Outcomes and Women’s Health Registry

The MSBase Pregnancy, Neonatal Outcomes and Women’s Health Registry began collecting detailed maternal and neonatal health outcomes on 10 May 2020.^21^ New data fields include assisted reproductive technology method, pregnancy outcome, obstetric/maternal complications, delivery method, reason for termination/miscarriage, congenital abnormality, birthweight, sex, and breastfeeding data.

Data are collected from 45 countries spanning the northern parts of Africa, North and South America, Asia, Australasia, Europe, and the Middle East.

### Study ethics

The Registry is integrated within the MSBase Registry (registered within the WHO International Clinical Trials Registry Platform ID ACTRN12605000455662). The MSBase Registry has ethics approvals or exemptions granted by each participating site’s human research ethics committee. All participants provide informed consent for their de-identified clinical data to be shared with researchers.

### Data acquisition and study population

Data were collected during routine outpatient neurology visits and recorded by neurologists or nurses in real-time and therefore reflect real-world clinical care. Data are collected from women of childbearing age with relapse-onset MS (ROMS), progressive-onset MS (POMS), radiologically isolated syndrome (RIS) and NMOSD (both probable and definite).

### Definitions

Any woman of **childbearing age** (18–45 years) actively followed in the MSBase Registry during the observation period was included in the present analysis. Women were classified as **actively followed** in our registry if they had at least one clinical visit during our **observation period** between 10 May 2020 (registry launch) and 1 August 2024 (date of data extract). A **completed pregnancy** refers to any pregnancy that has ended with an outcome reported encompassing live births, miscarriages, terminations, ectopic pregnancies and neonatal deaths.

### Statistical methods

We report summary statistics for demographic, clinical, treatment, pregnancy, birth, and neonatal outcomes data collected to-date. All variables were assessed for normality using the Shapiro-Wilk normality test, and reported as means and standard deviations (SD) or as medians and interquartile ranges (IQRs) as appropriate. Categorical data are reported as numbers and percentages.

## Data availability statement

MSBase is a data processor, warehousing data from individual principal investigators who agree to share their datasets on a project-by-project basis. Data access to external parties can be granted at the sole discretion of each MSBase Principal Investigator (the data controllers), who will need to be approached individually for permission. To make a request for data access, please contact the corresponding author for instructions.

## Results

### Patient and pregnancy characteristics

The MSBase Pregnancy, Neonatal Outcomes and Women’s Health Registry^21^ actively followed 20,850 women of childbearing age between 10 May 2020 and 1 August 2024. Of these 20,850 women, 20,539 had a diagnosis of MS, 40 had a diagnosis of radiologically isolated syndrome (RIS), and 271 had a diagnosis of NMOSD. These women had an average age of 37.3 years (SD 7.1 years) at data extract, with 2411 women now aged between 45 and 49 years. The median follow-up duration for this cohort in the MSBase Registry was 5.25 years (IQR: 1.9, 9.9). The median disease duration at data extract was 8.5 years (IQR: 4.3, 14.0) for women with MS, and 7.2 years (IQR: 2.9, 11.3) for women with NMOSD.

**Figure 1.**
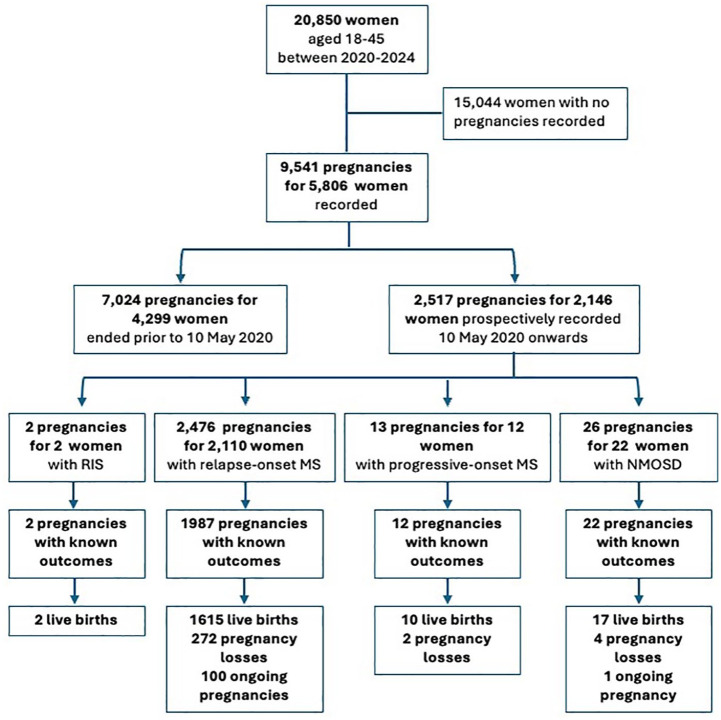
Consort flow diagram of study inclusion showing the number of pregnancies prospectively recorded, stratified by disease phenotype: radiologically isolated syndrome (RIS), relapse-onset MS (including secondary progressive MS), progressive-onset MS, and neuromyelitis optica spectrum disorder (NMOSD).

**Figure 2.**
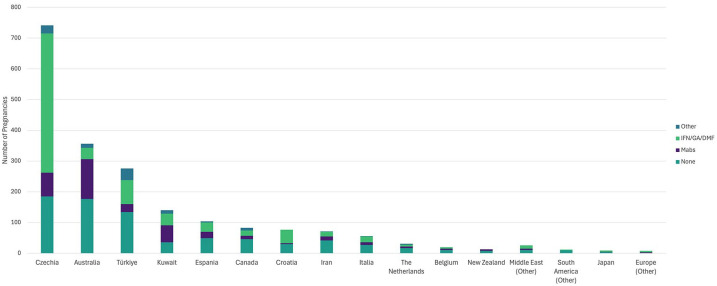
Number of pregnancies prospectively recorded by country, or region if fewer than 10 captured. This figure further displays the differences in regional peripartum management, showing the number of disease-modifying therapy (DMT) exposures during pregnancy, categorized by DMT type: none; interferons, glatiramer acetate or dimethyl fumarate (IFN/GA/DMF); monoclonal antibodies (Mabs, including: natalizumab, ocrelizumab, ofatumumab, rituximab, alemtuzumab); and all other therapies.

We recorded 9541 pregnancies for 5806 (27.8%) of the women followed during the observation period (see Figure 1). Of these, 7024 pregnancies for 4299 women were completed prior to our registry launch, and 2517 (26.4%) pregnancies for 2146 women were captured from 10 May 2020 onwards. The contribution by country to the registry is displayed in Figure 2.

Of the 2146 women who had pregnancies recorded in the newly launched Registry; 640 women had pregnancies both prior to and after Registry launch, and 1,507 women had pregnancies only after pregnancy registry launch. A total of 1820 (84.8%) women had a single pregnancy, 285 (13.3%) had two pregnancies, and 41 (1.9%) women reported three or more pregnancies during the observation period. The mean maternal age at the last menstrual period (LMP) was 32.3 years (SD 4.7 years), with a median expanded disability status scale (EDSS) score of 1.5 (IQR: 1, 2) within 12 months of the LMP. The median follow-up post-pregnancy was 1.7 years (IQR: 0.8, 2.6 years; range: 0.1–4.2 years). Detailed, per-phenotype, patient characteristics at each pregnancy are summarized in Table 1.

**Table 1.** Patient characteristics at each prospectively recorded pregnancy with known outcomes.

	ROMS, *n* = 1987	POMS, *n* = 12	RIS, *n* = 2	NMOSD, *n* = 22
**Age at pregnancy, years (mean, SD)**	32.3 (4.6)	36.8 (5.0)	31.5 (0.5)	28.9 (5.7)
**range**	18–45.5	27.8–43	31–31.8	20.8–43.5
**Disease duration at pregnancy (median, IQR)**	6.2 (3.1, 10.3)	5.4 (4.1, 7.7)	-	3.4 (0.7, 5.9)
**range**	0–30.9	0–15.1	-	0–20.2
**EDSS within 12** **m prior to pregnancy** ^a^	1.5 (1, 2)	3.5 (3, 5.5)	0	1 (0, 2)
**range**	0–7	2–7.5	0	0–3.5
**BMI within 12** **m prior to pregnancy (median, IQR)** ^b^	23.5 (21.2, 26.2)	NA	NA	33.2
**range**	17.0–50.9			
**ART use**				
IVF Ovulation drugs Other None Not reported	44 (2.2%) 2 (0.1%) 1 (0.05%) 528 (26.6%) 1,406 (70.8%)	1 (8.3%) - - 3 (25%) 8 (66.7%)	- - - 2 (100%) -	1 (4.5%) - - 12 (54.5.2%) 9 (41%)

ROMS: relapse-onset MS; POMS: progressive-onset MS; RIS: radiologically isolated syndrome; NMOSD: neuromyelitis optica spectrum disorder; SD: standard deviation; IQR: interquartile range; m: months; IVF: in vitro fertilization; IUI: intrauterine insemination; NA: not available.

a Data available for 1738 ROMS pregnancies; 8 POMS pregnancies; 2 RIS pregnancies; 16 NMOSD pregnancies.

b Data available for 102 ROMS pregnancies and 1 NMOSD pregnancy.

We had limited ascertainment of several demographic factors for the 2,146 women with prospectively reported pregnancies. Educational status was available for 684 (31.9%) of women. Of these 57 (8.3%) had completed elementary school, 194 (28.4%) had completed high school, and 433 (63.3%) had completed a tertiary degree. Employment status was available for 660 (30.7%) of women. Of these women, 441 (66.8%) were employed, 45 (6.8%) were unemployed, 83 (12.6%) performed domestic duties, 90 (13.6%) were students, and 1 (0.15%) reported being on a disability support pension. Smoking status was only available for 99 (4.6%) of women, of which 15 (15.1%) were current smokers, 17 (17.2%) were past smokers, and 67 (67.7%) were never smokers. We recorded 592 pregnancies with known outcomes for which assisted reproductive therapy (ART) data were available. Of these, 543 (91.7%) did not use ART; 46 conceived using in vitro fertilization (IVF), two used ovulation drugs and one used other methods.

### Pregnancy outcomes

As of 1 August 2024, 1922 (76.4%) of pregnancies were completed, 101 (4%) pregnancies were ongoing. 494 (19.6%) pregnancies did not have outcome data available beyond the date of the last menstrual period and were considered lost to follow-up. Of reported pregnancy outcomes 1464 (76.2%) of pregnancies resulted in full-term births, 180 (9.4%) were preterm births, totaling 1644 (85.5%) live births. 208 (13.7%) of pregnancies resulted in miscarriage, and 55 (2.9%) in termination. Six (0.3%) neonatal deaths and 9 (0.5%) ectopic pregnancies were reported. Of the total 1644 live births reported in our registry, birthweights were available for 413 term babies with a median weight of 3260 g (IQR: 3000, 3590). 918 (55.8%) babies were breastfed of which 150 (16.3%) reported exclusive breastfeeding for a median 3 months (IQR: 1, 5 months; range: 0–6 months). For detailed pregnancy outcomes by disease phenotype, see Table 2. Most pregnancies were singletons, with only 35 twin pregnancies reported. Of the twin pregnancies, 21 were born at term, nine preterm, four miscarriages, and one elective termination were also reported.

**Table 2.** Pregnancy and neonatal outcomes by disease phenotype for pregnancies with known outcomes.

	ROMS, *n* = 1,987	POMS, *n* = 12	RIS, *n* = 2	NMOSD, *n* = 22
**Pregnancy outcome**, *n* **(%)**				
Ongoing pregnancy	100 (5%)	-	-	1 (4.5%)
1^st^ Trimester	7			
2^nd^ Trimester	36			
3^rd^ Trimester	57			1
Term pregnancy (>= 37w) Term pregnancy (cong. ab) Preterm pregnancy (<37w) Preterm pregnancy (cong. ab) Live births total Neonatal Death^a^ Miscarriage (<20w) Miscarriage (>= 20w) Elective termination Ectopic pregnancy	1437 (72.3%) 2 (0.1%) 173 (8.7%) 3 (0.15%) 1615 (81.3%) 5 (0.3%) 202 (10.2%) 3 (0.2%) 53 (2.7%) 9 (0.5%)	9 (75%) - 1 (8.3%) - 10 (83%) - 1 (8.3%) - 1 (8.3%) -	1 (50%) - 1 (50%) -2 (100%) - - - - -	15 (68.2%) - 2 (9.1%) - 17 (85%) 1 (4.5%) 2 (9.1%) - 1 (4.5%) -
**Delivery mode**, *n* **(%)**^b^ Elective Cesarean Emergency Cesarean Vaginal Delivery Vaginal Delivery (assisted) Not reported	*n* **=** **781** 226 (28.9%) 78 (10%) 423 (54.2%) 59 (7.6%) 834	*n* **=** **5** 2 (40%) - 3 (60%) - 5	*n* **=** **2** - 1 (50%) 1 (50%) - -	*n* **=** **9** 4 (44.4%) - 4 (44.4%) 1 (11.1%) 8
**Baby sex**^b^, *n* **(%)** Female Male	*n* **=** **728** 351 (48.2%) 377 (51.8%)			
**Weight**^b, c^, *n* **(%)** kg (median, IQR)	*n* **=** **407 (28.2%)** 3.3 kg (3 kg, 3.6 kg)			
**range**	1.9 kg–5.5 kg			
**Pregnancies DMT exposed**, *n* **(%)**^b^	1228 (61.8%)	5 (41.7%)	0 (0%)	4(19%)
**Live birth pregnancies DMT exposed**, *n* **(%)**	1006 (62.3%)	3 (30%)	0 (0%)	4(23.5%)
**Breastfed**, *n* **(%)**^b^	903 (55.9%)	3 (30%)	2 (100%)	10 (58.8%)
**Breastfed exclusively**, *n* **(%)**^d^	146 (16.2%)	2 (67%)	1 (50%)	1 (10%)

ROMS: relapse-onset MS; POMS: progressive-onset MS; RIS: radiologically isolated syndrome; NMOSD: neuromyelitis optica spectrum disorder; SD: standard deviation; IQR: interquartile range; m: months.

a MS neonatal deaths occurred in 2 unexposed pregnancies, 2 pregnancies exposed to interferon-beta-1a-SC, 1 pregnancy exposed to natalizumab. NMOSD pregnancy was exposed to rituximab.

b Percentage based on known outcomes for live births.

c Term pregnancies only. Too few data points to report for POMS, RIS, and NMOSD pregnancies individually.

d Percentage of breastfed babies.

The majority of women had unassisted (54.1%) or assisted (7.5%) vaginal births. Elective cesarean sections comprised 29.1% of delivery methods, most often performed in Iran (57.1%), Turkey (50.9%), Italy (46.3%), and Australia (37%). Birth complication data were reported for 614 (31.9%) completed pregnancies. Of these 535 (87.1%) reported no complications. The most common complications were gestational diabetes (*n* = 16) and pre-eclampsia or pregnancy-induced hypertension (*n* = 10). Antepartum hemorrhage was reported in five women. Additional complications reported included: growth restriction, large for gestational age, low birth weight, oligohydramnios, polyhydramnios, premature membrane rupture, placenta previa, placental abruption, and others.

### Disease-modifying therapy exposures

In the 6 months prior to conception, 506 (25%) pregnancies with known outcomes had no DMT exposure and 1517 (75%) were DMT-exposed, of which 282 (18.6%) discontinued within the 6 months prior to conception with a median 31 days washout (IQR: 2 day, 97 days). Disease-modifying therapy (DMT) exposure in-pregnancy was reported for 1228 (61.8%) pregnancies in women with ROMS, 5 (41.7%) pregnancies in women with POMS, and 4 (19%) NMOSD pregnancies (Table 2). DMT exposure trends varied by country (Figure 2). The interferons, glatiramer acetate, and dimethyl fumarate were most commonly used in pregnancy with 37.8% of pregnancies exposed to these drugs. In-pregnancy monoclonal antibody infusions were recorded in 18.1% of pregnancies.

**Figure 3.**
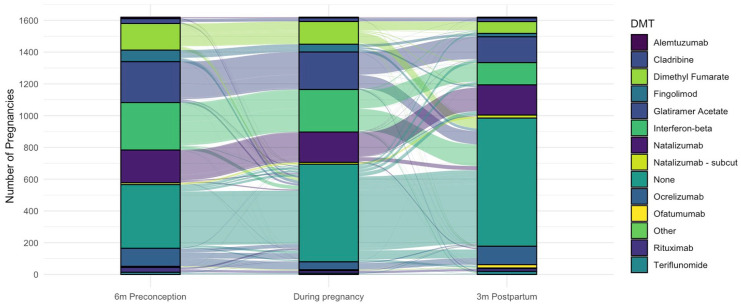
Frequencies and patterns of disease-modifying therapy (DMT) exposure 6 months preconception, during pregnancy (for any duration) and in the first 3 months postpartum in women with relapse-onset MS. DMTs with fewer than five exposures grouped together in the “other” category.

Of ROMS pregnancies, 1006 (62.3%) of live birth (term and preterm) pregnancies received DMT during pregnancy. The median length of DMT exposure in pregnancy was 42 days (IQR: 25, 218; range: 1–290). Figure 3 summarizes the DMT exposure trends in women with ROMS who had live births from the 6 months prior to conception through to 3 months postpartum. The per-DMT exposure durations for this cohort are summarized in Table 3. Postpartum, 811 (50.2%) ROMS women with live births resumed DMT use within 3 months (Figure 3, Table 3). Whereas, 183 (68%) women reporting miscarriages, terminations and other adverse pregnancy outcomes resumed DMT within 3 months of pregnancy completion.

**Table 3.** Disease-modifying therapy use associated with 1615 live births to women with relapse-onset MS.

Disease-modifying therapy	Number of discontinuations within 6 months of LMP	Days discontinued prior to LMP, median (IQR; range)	Gestational exposures *n* (% total on drug)^a^	Days exposure, median (IQR; range)	Number of DMT restarts within 3 months after delivery	Days to restart within 3 months, median (IQR; range)
Azathioprine	-	-	1 (100%)	32		
Interferon-beta-1a IM	8	0 (1, 0; 0–14)	51 (86%)	30 (15, 44; 1-286)	25	0 (0, 50; 0–91)
Interferon-beta-1a SC	27	21 (0, 73; 0–179)	117 (91.4%)	37 (28, 58; 1–288)	14	0 (0, 0; 0–45)
Interferon-beta-1b	4	5 (0, 74; 0–139)	20 (80%)	54 (24, 270; 1-286)	71	25 (0, 58; 0–90)
Pegylated Interferon-beta	13	2 (0, 16; 0–141)	53 (95%)	31 (24, 73; 1–286)	30	16 (0, 46; 0–86)
Glatiramer Acetate	39	5 (0, 31; 0–149)	211 (89.4%)	41 (27, 231; 1–290)	163	0 (0, 43; 0–91)
Teriflunomide	6	77 (35, 128; 31–146)	7 (88%)	35 (31, 66; 22–85)	20	52 (16, 69; 0–88)
Dimethyl Fumarate	40	19 (0, 54; 0–152)	135 (94.4%)	30 (17, 43; 1–284)	74	48 (6, 70; 0–91)
Diroximel Fumarate	-	-	-	-	1	82
Fingolimod	25	65 (30, 89; 0–183)	48 (98%)	27 (13, 46; 1–92)	21	35 (15, 64; 0–82)
Siponimod	-	-	-	-	1	5
Cladribine	6	128 (120, 153; 45–173)	1 (100%)	124	23	0 (0, 0; 0–90)
Alemtuzumab	3	169 (167–181)	2 (100%)	191, 228	5	7 (0, 7; 0–31)
Natalizumab (IV)	19	16 (4, 30; 0–178)	174 (91%)	202 (40, 230; 1–251)	190	10 (0, 36; 0–89)
Natalizumab (SC)	1	19	12 (100%)	235 (202, 256; 87–283)	19	73 (51, 81; 0–90)
Ocrelizumab	69	71 (18, 115; 0–174)	29 (58%)	26 (2, 36; 1–146)	116	26 (3, 56; 0–91)
Ofatumumab	1	0	3 (100%)	1, 10, 11	21	29 (21, 56; 2–83)
Rituximab	10	97 (69, 131; 45–142)	5 (100%)	26 (24, 43; 1-46)	17	0 (0, 0; 0–14)
Methotrexate	1	13	-	-	-	-
No DMT exposure	337	-	609	-	804	-

a Note not all clinicians reported in-pregnancy DMT stop dates. Data based on 869/1006 exposures with stop dates reported.

In the NMOSD cohort, six rituximab exposures were recorded in the 6 months prior to conception. Of these, two ceased therapy prior to conception with a washout of 2 and 4 months, respectively. Four rituximab exposures occurred during pregnancy, median gestation at DMT exposure was 26 days (IQR: 24, 43). Of these six pregnancies, five resulted in healthy, term deliveries, and one in neonatal death. One woman was exposed to eculizumab 2.5 months prior to conception; this pregnancy was aborted. No women with NMOSD restarted DMT within 3 months postpartum; however, five women resumed rituximab a median 271 days (IQR: 219, 287) after the completion of their pregnancies. We did not observe any perinatal exposures to satralizumab.

Of 918 breastfed babies, 296 (32.2%) were DMT-exposed during breastfeeding in the first 3 months postpartum. All breastfeeding mothers who resumed DMT within the first 3 months postpartum had ROMS. The median time to DMT reinitiation during this period was 2 days (IQR: 0, 43; range: 0–91). The DMTs resumed during breastfeeding included glatiramer acetate (*n* = 78), interferon-beta preparations (n = 62), natalizumab infusions (*n* = 60), ocrelizumab (n = 20), pegylated-interferon-beta (*n* = 17), dimethyl fumarate (*n* = 14), rituximab (*n* = 12), natalizumab subcutaneous injections (*n* = 10), ofatumumab (*n* = 7) and others (n = 16).

### Congenital abnormalities

In total, five pregnancies were reported with congenital abnormalities, three preterm pregnancies and two term pregnancies. Of the three preterm pregnancies, one had been exposed to glatiramer acetate for 38 days, another received rituximab at 43 days gestation, the third was not DMT-exposed. No specifics of the congenital abnormalities were reported for this group. One term pregnancy reporting a chromosomal abnormality had been exposed to dimethyl fumarate for 37 days. This mother had a history of prior pregnancies, one of which had also resulted in the birth of a child with congenital abnormalities. The other term pregnancy with congenital abnormality was not DMT-exposed.

## Discussion

Family planning and pregnancy decisions are key considerations in the management of women with MS and NMOSD. In the era of highly effective disease-modifying therapies knowledge gaps remain, including best management strategies through pregnancy and whether there are fetal risks associated with DMT exposure *in utero*.

In this first report from the newly launched prospective MSBase Pregnancy, Neonatal Outcomes and Women’s Health Registry, we report 101 ongoing pregnancies, and pregnancy outcomes for 1922 completed pregnancies, including 21 completed pregnancies for women with NMOSD.

Over 85% of pregnancies with known outcomes resulted in live births, in line with other recently reported studies.^22^ In our registry, 9% of births were preterm births, 10% of pregnancies were miscarried, reassuringly in line with data from German MS Pregnancy Registry,^23^ and in line with global rates in the general population as per the World Health Organization.^24,25^

Interestingly, 75% of pregnancies were DMT exposed in the 6 months prior to conception, decreasing to 62% of pregnancies during gestation, an overall lower percentage of DMT use than in the German pregnancy registry that reported 76% exposure at pregnancy.^23^ Ours may be a slight underestimate as we measured the date of last infusion for monoclonal antibodies, but did not estimate the duration of effect of these drugs. We know, for example that infusional anti-CD20 monoclonal antibodies have a durable effect, protecting against disease activity for many months into pregnancy and potentially beyond.^6^ The recent study reporting pregnancy outcomes from the Roche pharmacovigilance study for example defined exposure to ocrelizumab if the last infusion was within 3 months of conception.^22^ Given that here we assessed numerous infusional therapies with varying mechanisms of action, here we determined to use a consistent definition for all. Postpartum, only 50% of our study population with live births had restarted DMT use within the first 3 months of delivery. Most women resumed the use of the same DMT as they had used preconception or during pregnancy, although we did observe a low level of DMT switch. The median exposure to DMT during pregnancy was 42 days. This has increased marginally since our previous 2019 report showing a median exposure of 30 days,^26^ indicating that many women still discontinue DMT upon pregnancy confirmation. However, we did see a marked increase in the duration of natalizumab exposure, median 202 days (28.9 weeks) in the present study, as compared with 24 days in our previous report.^26^ This reflects our increased understanding of maternal and fetal outcomes in babies exposed to natalizumab in utero,^12^ and comfort with using natalizumab through to the end of the second trimester to minimize risk of in-pregnancy disease rebound upon withdrawal.^4,7^

We observed pregnancy management varied among countries. Australian and Kuwaiti clinicians were most likely to manage pregnancies with monoclonal antibodies, whereas Europeans were most likely to use interferons, glatiramer acetate or dimethyl fumarate to manage pregnancy. These differences likely reflect local legislative and therapeutic agency indications for these drugs. While still relatively new, we saw the growing use of ocrelizumab, ofatumumab and subcutaneous natalizumab use perinatally.

While we did not have sufficient weight or other biometric data to analyze neonatal outcomes by DMT exposure, regardless, no overt safety signals were observed. Previous studies have not reported overt differences in outcomes of babies born to mothers with MS, and non-MS controls.^27^ Furthermore, studies by other groups have not reported concerning safety signals for the newer DMT including natalizumab^12,28^ and ocrelizumab.^22^

Few studies to-date have reported obstetric outcomes for women with NMOSD.^29–31^ Here we report 17 (81%) live births and one neonatal death (4.8%) for 21 completed pregnancies in women with NMOSD. Six pregnancies, including the neonatal death, were exposed to rituximab. An analysis of a 153 rituximab-exposed pregnancies reported one neonatal death at 6 weeks.^32^ In the general population, across Europe, North America, and Australasia, neonatal deaths occur in 2–3 per 1000 live births.^33^ Here we reported six neonatal deaths across our MS and NMOSD cohorts, marginally higher than the expected five given our cohort size. However, our per-DMT sample size is too small to draw any causal conclusions.

A key strength of our Registry is the unified, prospective, multicenter, international dataset that we have been able to accumulate, with over 2000 pregnancies from women with MS, RIS, and NMOSD recorded over a 4-year period. With time and increasing data collection, we will be able to compare pregnancy and neonatal outcomes across various regions and accumulate real-world evidence for safety signals. Our registry has further worked with the BigMS network to align key data fields, so that we will be able to collaboratively report on safety signals across registries in the future. We will therefore continue to contribute critical evidence for MS management and obstetric care.

We do, however, have several limitations. Our neonatal and pregnancy outcome data are self-reported; therefore, we are unable to confirm outcomes with obstetricians, pediatricians, or other healthcare professionals. Further, details of congenital abnormalities for 4/5 pregnancies were unavailable, and pregnancy complications were underreported. We continue to work with our network to ensure the most accurate acquisition of this important data as we move forward. Another limitation of our registry is that a large number of pregnancies, almost 20%, were lost to follow-up. We have now initiated three-monthly reminders to our network to follow up on known pregnancies, so that we can reduce the number lost to follow-up. Furthermore, the international nature of our database, differing pediatric practice, and legal frameworks means that we are unable to collect neonatal outcomes beyond 12 months of age. To better understand the impacts of DMT exposure in children born to mothers with MS, we will eagerly anticipate results from national registries such as the French RESPONSE study that will be collecting these outcomes out to 6 years.^10^

## Conclusion

This first report from the international MSBase pregnancy registry shows that an increasing number of pregnancies are conceived on monoclonal antibody therapies, confirming increased confidence in their use in the peri-conception period. Reassuringly our data do not show any safety issues at present. However, in an era where pregnancies are increasingly exposed to DMT use and with the growing number of DMT available, it is important to continue to collect data to monitor for fetal and maternal serious adverse events in real-world databases.
